# Phytotoxic dioxolanones are potential virulence factors in the infection process of *Guignardia bidwellii*

**DOI:** 10.1038/s41598-017-09157-6

**Published:** 2017-08-21

**Authors:** Iris Buckel, Lars Andernach, Anja Schüffler, Meike Piepenbring, Till Opatz, Eckhard Thines

**Affiliations:** 1 Johannes Gutenberg-University, Mikrobiologie und Weinforschung, Institute of Moleculare Physiology, Johann-Joachim-Becherweg 15, 55128 Mainz, Germany; 2 Johannes Gutenberg-University, Institute of Organic Chemistry, Duesbergweg 10-14, 55128 Mainz, Germany; 3 Institut für Biotechnologie und Wirkstoff-Forschung gGmbH (IBWF), Erwin-Schrödinger-Str. 56, 67663 Kaiserslautern, Germany; 4 Goethe University Frankfurt, Department of Mycology, Max-von-Laue-Str. 13, 60438 Frankfurt am Main, Germany

## Abstract

Phytotoxic dioxolanones from *Guignardia bidwellii* can be described as potential virulence factors which cause the formation of lesions upon an infection by *G. bidwellii*. The toxin guignardic acid was found *in planta* of *G. bidwellii*-infected *Vitis vinifera* leaves, whereas no phytotoxic dioxolanones were detected in uninfected leaf material. Secondary metabolism analyses of further phytopathogenic fungi from the genus *Guignardia* led to the observation that all species investigated can produce the phytotoxins known from *G. bidwellii*. In addition to these studies, it was demonstrated that phenguignardic acid is biosynthetically derived from two molecules of phenylalanine and that phenylalanine is a key precursor in the biosynthesis of the two other phytotoxins – alaguignardic acid and guignardic acid.

## Introduction

*Guignardia bidwellii* (Ellis) Viala & Ravaz (currently valid name *Phyllosticta ampelicida* (Engelm.) Aa; Botryosphaeriales, Ascomycota) is the causal agent of grape black rot^1^. This disease can cause substantial losses of grapes in areas with humid summers^2^. Previous studies of submerged fermentations of *G. bidwellii* led to the identification of nine secondary metabolites, five of which are phytotoxic^3, 4^. The metabolites contain a dioxolanone moiety and are presumably biosynthesised from transaminated amino acids, such as phenylalanine, valine, tyrosine and alanine. This is supported by the fact that supplementation experiments with phenylalanine led to an enhanced biosynthesis rate of one of the phytotoxic dioxolanones. In addition, a cryptic NRPS-like gene called pgnA was identified recently in *Aspergillus terreus* and its heterologous expression in *Aspergillus nidulans* confirmed that this gene is solely responsible for the biosynthesis of phenguignardic acid^5^. Sun *et al*.^5^ proposed its formation by linking two phenylpyruvic acids. The toxins are non-host specific and a structure activity relationship indicated that a free carboxyl group appears to be required for the phytotoxic activity^4^.

The secondary metabolites are believed to be involved in the pathogenicity of *G. bidwellii* and can be described as potential virulence factors, due to the fact that the application of the pure compounds onto the host tissue causes specific disease symptoms^6^. Additional studies should be conducted to corroborate this hypothesis. It is necessary to analyse whether the toxins can be found upon infection by the fungus *in planta*. Furthermore, the questions whether other phytopathogenic species of the *Guignardia* relationship can produce the phytotoxic dioxolanones and whether apathogenic species are toxin-free need to be addressed.

These questions were considered in the present study. As for the question whether the toxin can be identified *in planta* after infection by *G. bidwellii*, infected and non-infected leaf material of *Vitis vinifera* was analysed for the presence of phytotoxic dioxolanones. Three phytopathogenic species and one biotrophic endophyte of the genus *Guignardia* were cultivated and the production of secondary metabolites was analysed to see whether these fungi can produce the phytotoxic principles. In addition, the precursors for the biosynthesis of the phytotoxic dioxolanones were elucidated by isotopic labelling.

## Results and Discussion

### Toxin detection *in planta*

**Figure 1:**
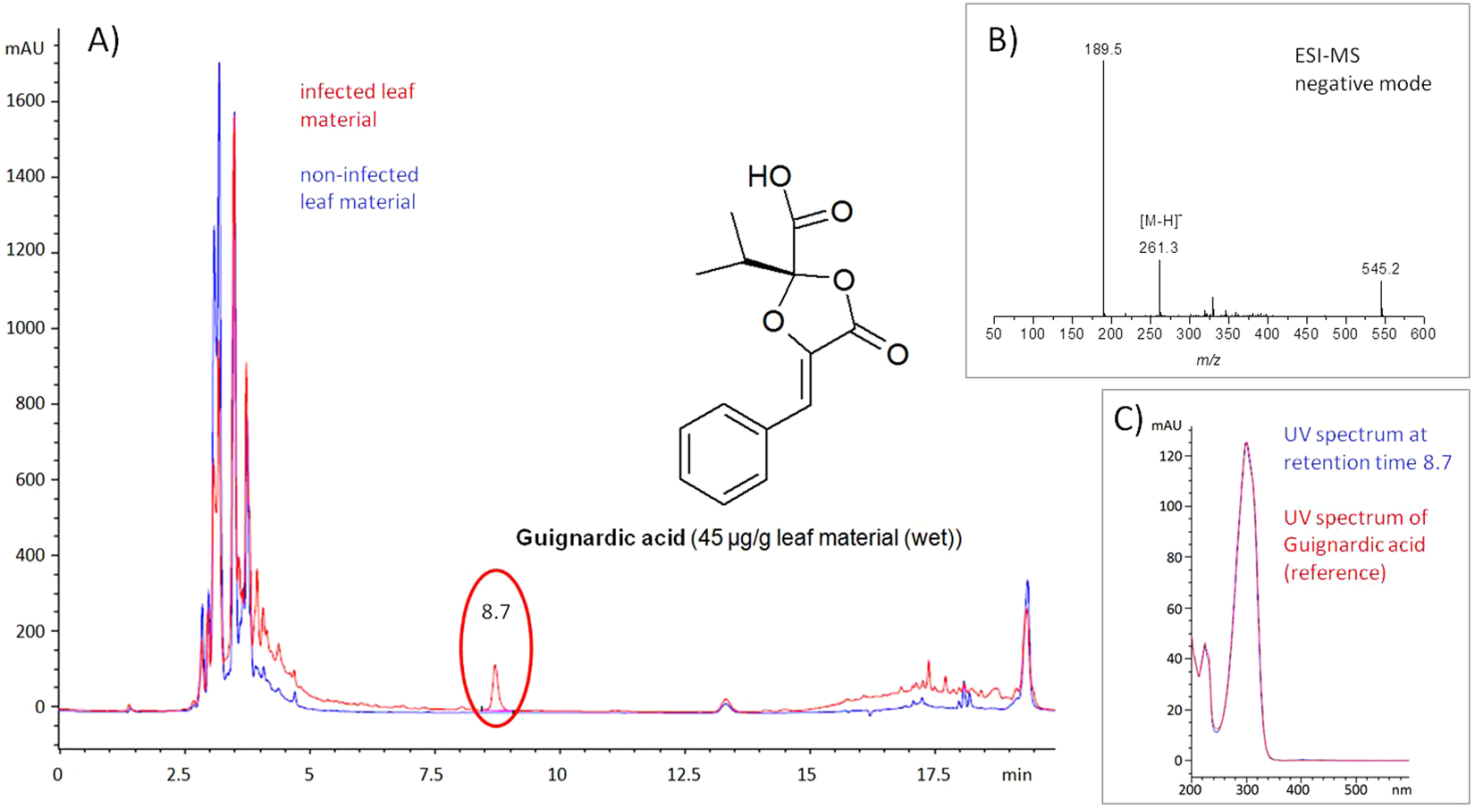
HPLC-MS analysis of extracts of *Guignardia bidwellii*-infected and non-infected leaves of *Vitis vinifera*. (**A**) The chromatograms at 300 nm of both infected (red) and non-infected (blue) samples were combined. The signal at a retention time of 8.7 min, detectable only in the infected sample, was identified by HPLC-MS as guignardic acid, a known phytotoxic secondary metabolite of *G. bidwellii*. (**B**) ESI-MS negative mode of the signal at 8.7 min corresponds to [M-H]^−^ of guignardic acid. (**C**) Comparison of the UV spectrum of the signal at 8.7 min (blue) with guignardic acid reference spectrum (red).

The production of phytotoxins *in planta* is an important requirement to assume the involvement of the toxin in pathogenicity^7^. In this study, both *G. bidwellii*-infected and non-infected leaf material was analysed by analytical HPLC after extraction. Comparison of both chromatograms (Fig. 1A) led to the detection of a signal at a retention time of 8.7 min in the infected sample (Fig. 1A, red), which was not present in the non-infected sample (Fig. 1A, blue).

The UV spectra recorded for this compound (Fig. 1C) resemble those of the phytotoxic dioxolanones known from *G. bidwellii* (local maxima around 230 nm and 295 nm^3, 4^). By isolation of the metabolite and HPLC-MS analysis the metabolite was identified as guignardic acid. This compound is the first member of the new class of secondary metabolites described^8^. Thus, the production of one of the toxins of the fungus during active growth *in planta* was proven. The detection of phytotoxins *in planta* needs highly sensitive methods of measurement, because most toxins are highly potent, therefore, only small quantities are required to cause symptoms of infection^9^. A concentration of 45 µg/g leaf material (wet) of guignardic acid was determined experimentally in the infected sample.

### Toxin production in other species of the genus *Guignardia*

Phytotoxins, described as virulence factors, are frequently characteristic for a whole genus^10^. Several phytopathogenic *Fusarium* species, for example, produce trichothecenes, which inhibit eukaryotic protein biosynthesis. If the biosynthesis of trichothecenes is impaired, virulence is reduced (refs 11, 12 and references therein). Isolates of the genus *Cercospora* produce cercosporin and the targeted disruption of its biosynthesis in *C. nicotianae* led to fewer necrotic lesions on inoculated tobacco leaves^13, 14^. Both groups of toxins are therefore regarded as virulence factors. Phytotoxic dioxolanones from *G. bidwellii* can also be considered as virulence factors. We hypothesise that closely related species produce similar compounds, so three further phytopathogenic species and one species reported as endophyte^15–18^ of the genus *Guignardia* were explored (Table 1).

**Table 1:** *Guignardia* species examined and the phytotoxic dioxolanones identified.

Organism	Life style	Secondary metabolites produced	Metabolite concentration [mg/l]
*G*. *aesculi* (Peck) V.B. Stewart (**CBS 756.70**)	phytopathogenic	phenguignardic acid	41.2
*G*. *musae* Racib. (**IMI 147360**)	phytopathogenic	guignardic acid	7.9
		phenguignardic acid	2.1
*G. vaccinii* Shear (**CBS 126.22**)	phytopathogenic	guignardic acid	208.7
		phenguignardic acid	19.9
*G. mangiferae* A.J. Roy (**CBS 123405**)	endophytic	guignardic acid	7.3

*G. aesculi*, *G. musae*, *G. vaccinii* and *G. mangiferae* were cultivated by submerged fermentation and analysed for the production of secondary metabolites. Metabolite concentrations are given in mg/l culture.

All species investigated produce one or more phytotoxic dioxolanones known from *G. bidwellii* in submerged cultures. Table 1 gives an overview of the secondary metabolites produced and their amount determined experimentally.

The fact that all *Guignardia* species examined produce dioxolanone-type secondary metabolites known from *G. bidwellii* supports the assumption that the toxins are virulence factors.

Interestingly, even *G. mangiferae* produces guignardic acid, although the fungus was isolated as a biotrophic endophyte^19^. A possible explanation could be that usually silent gene clusters responsible for phytotoxin production were activated within the artificial environment during fermentation. Another possibility could be that *G. mangiferae* produces the toxin even within the host, but the lesion formation is inhibited by the plant defence and, therefore, the fungus was classified as an endophyte^20^. Kuldau and Yates^21^ demonstrated that experimental or suboptimal conditions could stress the host and, therefore, the plant defence is weakened. This can result in disease caused by a usually asymptomatic fungus. Thus, the possibility that a fungus becomes pathogenic, although isolated as endophyte, cannot be ruled out^21^.

The study demonstrated that all *Guignardia* species investigated can produce phytotoxic dioxolanones. Therefore, these compounds can be described as potential virulence factors in the infection process.

### Elucidation of the precursor for the biosynthesis of phytotoxic dioxolanones

Phytotoxic dioxolanones from *G. bidwellii* have not been studied extensively to date^4^. Even though the genetic origin of phenguignardic acid was recently uncovered by the heterologous expression of a cryptic NRPS-like gene found in *Aspergillus terreus* in *Aspergillus nidulans* ^5^, the biosynthesis in *G. bidwellii* is not yet understood. Previous studies have led to the assumption that the compounds were built up from α-keto acids related to proteinogenic amino acids. An involvement of the keto-precursors to phenylalanine and valine in guignardic acid, two phenylalanines in phenguignardic acid, phenylalanine and alanine in alaguignardic acid and phenylalanine and tyrosine in guignardianone E and F is assumed^3, 4^.

Supplementation experiments with the precursor phenylalanine or with pyridoxal phosphate, the coenzyme of the amino acid metabolic pathway, were carried out during submerged fermentation of *G. bidwellii* and led to increased yields of phenguignardic acid^4^. These results support the theory that amino acids are biosynthetic precursors for the phytotoxic dioxolanones.

Supplementation experiments were performed with ^13^C-labelled phenylalanine in this study to verify this hypothesis.

**Figure 2:**
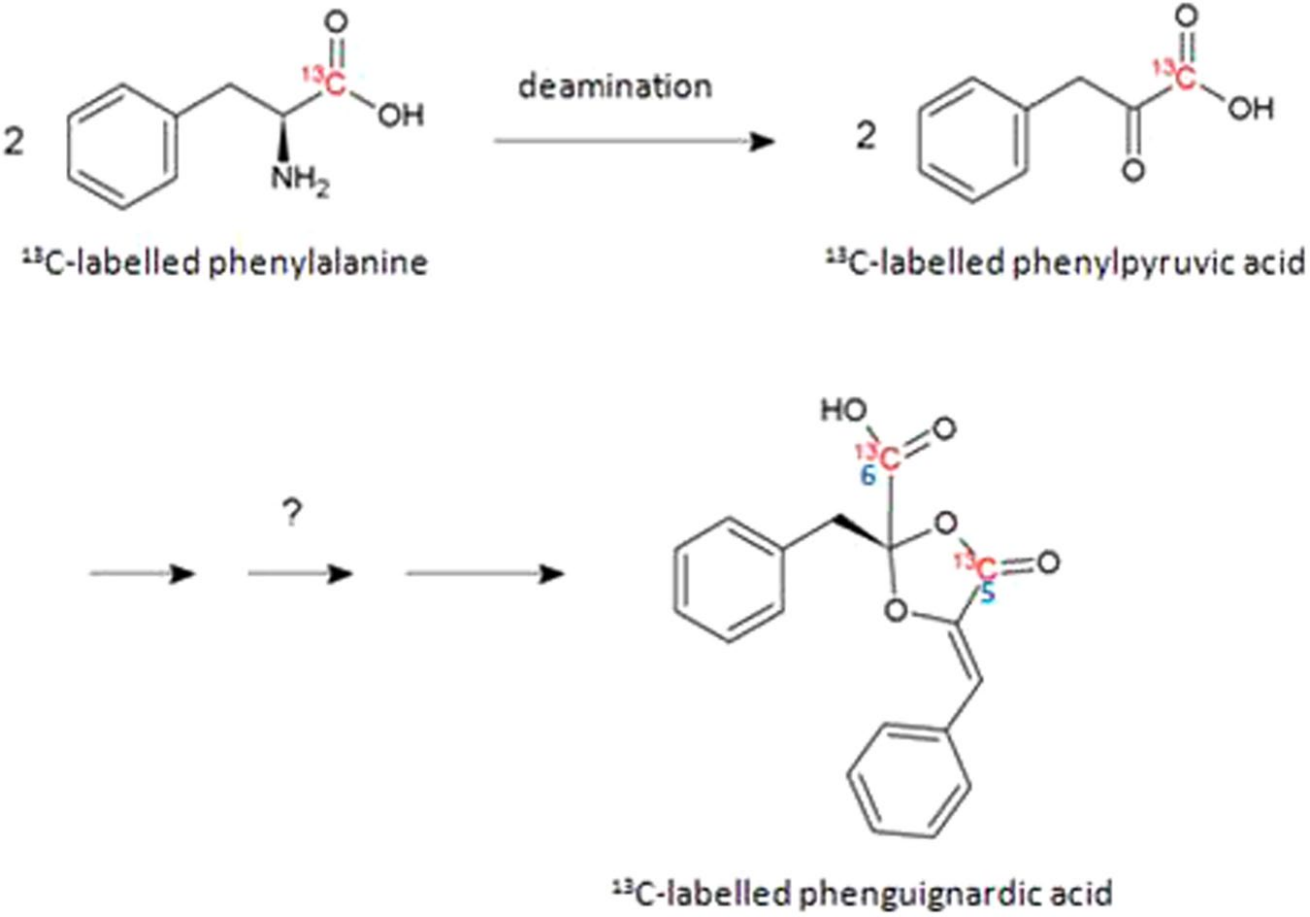
Hypothetical model of the biosynthesis of phenguignardic acid. Starting from two molecules of 1-^13^C-labelled phenylalanine, the amino group could be transferred and two molecules of phenylpyruvic acid could be generated. These molecules could fuse and form a dioxolanone moiety, whereby phenguignardic acid is formed.

If phenylalanine was the precursor for the biosynthesis of the toxins, an enrichment of ^13^C at the C-5 position (and, in addition, the C-6 position in phenguignardic acid) would be detectable. Figure 2 shows the hypothetical biosynthesis of phenguignardic acid.

After fermentation of *G. bidwellii* with supplemented ^13^C-labelled phenylalanine, the pure compounds alaguignardic acid, guignardic acid and phenguignardic acid were isolated by preparative HPLC and analysed by HPLC-MS and ^13^C NMR spectroscopy.

**Figure 3:**
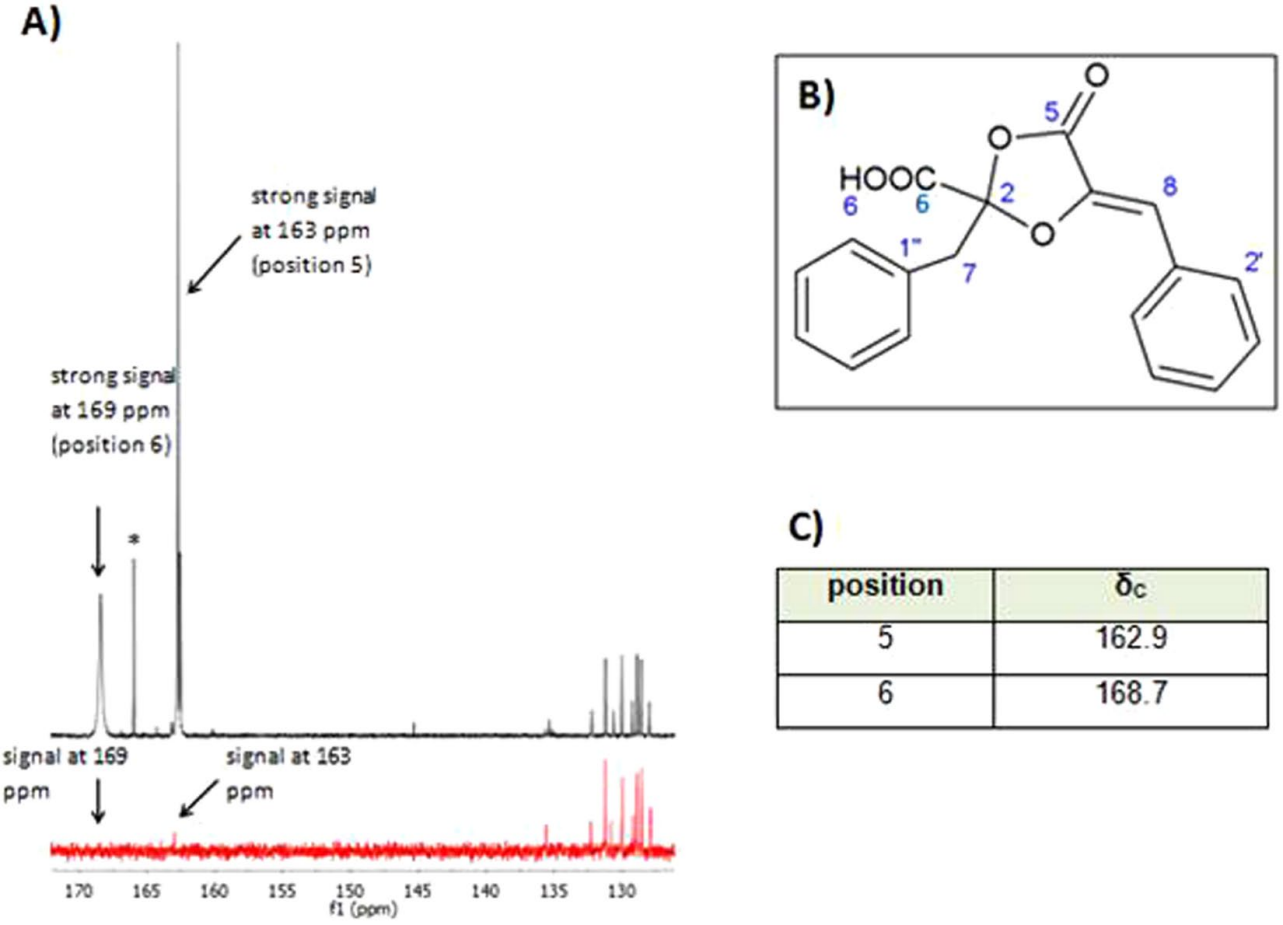
Spectrum of ^13^C NMR measurement of ^13^C-labelled and non-labelled phenguignardic acid with structural formula and ^13^C NMR Data. (**A**) Strong signals were detected at 163 ppm and 169 ppm (black) in the NMR spectrum of phenguignardic acid, whereas these signals had lower intensity in the NMR spectrum of phenguignardic acid without labelling (red). This indicates an enrichment of ^13^C at the C-5 and C-6 position in phenguignardic acid. (**B**) Structural formula of phenguignardic acid. (**C**) Segment of ^13^C NMR data of phenguignardic acid^3^.

Strong phenguignardic acid signals at 163 and 169 ppm could be detected in the ^13^C NMR spectra of the labelled sample (Fig. 3A, black). By contrast, these signals had a lower intensity in the unlabelled sample of phenguignardic acid (Fig. 3A, red), while the remaining signals in the range of 125 and 135 ppm have equal intensity in both spectra. Compared with NMR data from Molitor *et al*.^3^ and the structural formula of phenguignardic acid (Fig. 3B,C), the labelled carbon atoms are located at the C-5 and C-6 position in phenguignardic acid. This agrees with the proposed biosynthetic pathway shown in Fig. 2, as well as that proposed by Sun *et al*.^5^.

**Figure 4:**
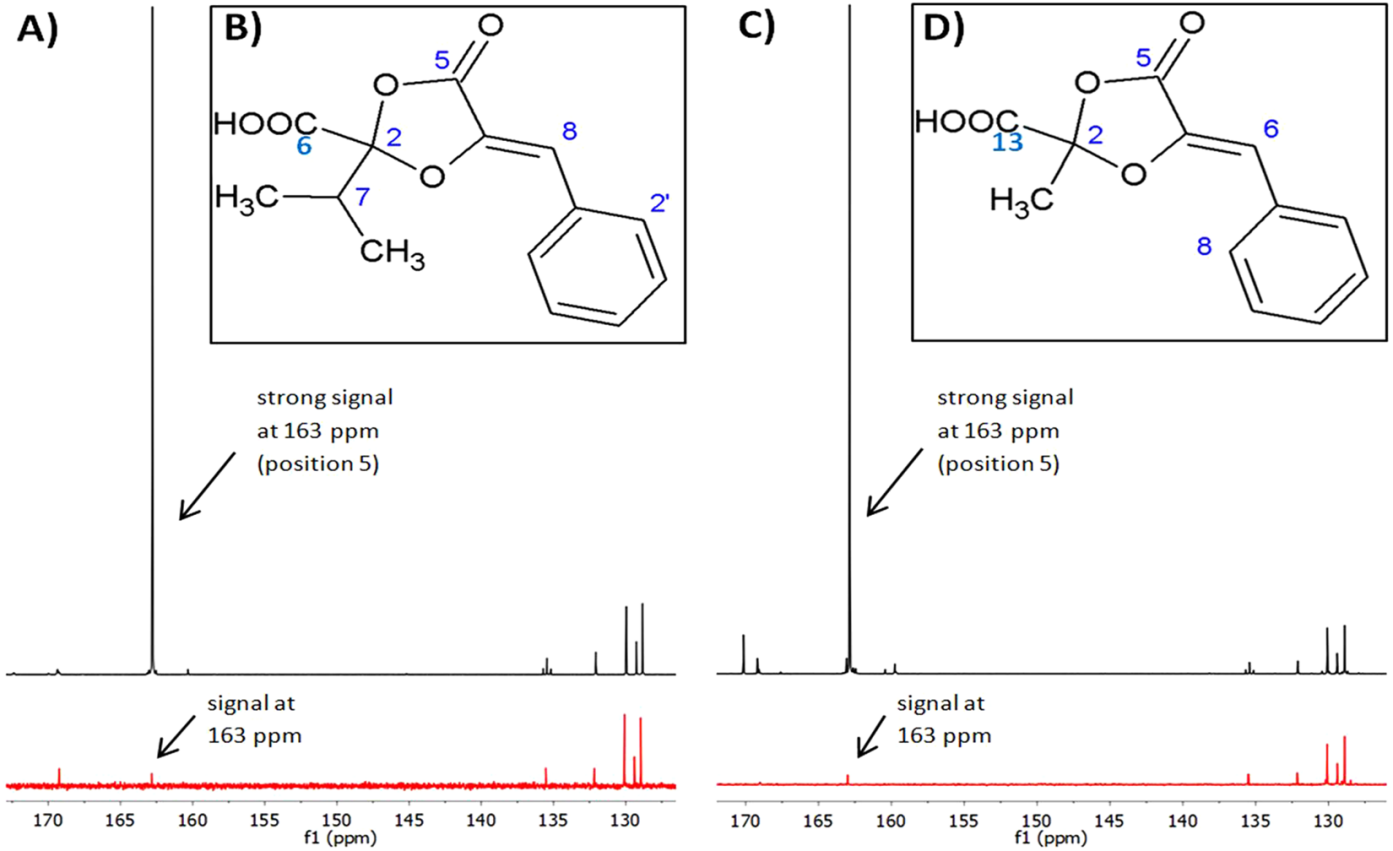
Spectra of ^13^C NMR measurement of ^13^C-labelled and non-labelled guignardic acid and alaguignardic acid with structural formulas. (**A**,**C**) A strong signal was detected at 163 ppm (black) in the NMR spectrum of guignardic acid, respectively, alaguignardic acid, whereas this signal with lower intensity was detectable (red) within the NMR spectrum of the compounds without labelling. This is significant for an enrichment of ^13^C at the C-5 position in guignardic acid, respectively, alaguignardic acid. (**B**) Structural formulae of guignardic acid. (**D**) Structural formula of alaguignardic acid.

The investigation of the ^13^C-labelled samples of guignardic acid and alaguignardic acid by NMR spectroscopy led to a strong increase of the signal at 162 ppm in both samples (Fig. 4A,C, black).

Only signals with lower intensity at this position could be found in the unlabelled samples of the compounds (Fig. 4A,C, red). Similar to phenguignardic acid, the signals in the range of 125 and 135 ppm have equal intensity in both spectra. The labelled carbon atom could be identified as C-5 by comparison with NMR data from Rodrigues-Heerklotz *et al*.^8^ and Buckel *et al*.^4^ and the structural formula of guignardic acid and alaguignardic acid (Fig. 4B,D).

The results of these studies verify the hypothesis that two molecules of phenylalanine are required for the biosynthesis of phenguignardic acid. Furthermore, an involvement of phenylalanine as an essential module in all compounds from *G. bidwellii* investigated was proven. These results support the proposed biosynthesis via an NRPS-like gene by Sun *et al*.^5^.

This is an important step towards the elucidation of the biosynthesis of phytotoxic dioxolanones in *G. bidwellii*. The next step is to characterize their genetic origin. Even if Sun *et al*.^5^ found the NRPS-like gene in *Aspergillus terreus* responsible for the biosynthesis of phenguignardic acid, nothing is known to date regarding the genus *Guignardia* and the other dioxolanones, such as guignardic acid and alaguignardic acid.

The phytotoxic secondary metabolites from *G. bidwellii* can be described as potential virulence factors due to the facts that the toxin guignardic acid is detectable in *G. bidwellii*-infected leaves of *V. vinifera* and all species of the genus *Guignardia* investigated can produce these phytotoxic dioxolanones. Therefore, these compounds seem to play a crucial role in the infection process of *G. bidwellii*. The secondary metabolites belong to a new class of natural products and their biosynthesis in *G. bidwellii* has still not been understood. It could be proven by feeding experiments with ^13^C-phenylalanine that all phytotoxic dioxolanones incorporate at least one phenylalanine. These results form a reliable base for further investigation into the pathogen-host-interaction between *G. bidwellii* and *V. vinifera* and can help to develop new defence strategies, even for applications in ecological vineyards.

## Materials and Methods

### General experimental procedures

Compounds were analysed by HPLC (Agilent 1100 Series) equipped with an Eclipse XDB Phenyl-column (3 × 150 mm; 3.5 µm, Agilent, Waldbronn, Germany) at 40 °C and a flow rate of 0.7 ml min^−1^. The elution gradient was composed of H_2_O + 0.1% v/v phosphoric acid and acetonitrile. Compounds were detected via UV at 300 nm. The quantification of guignardic acid and phenguignardic acid was performed using an external standard. A calibration curve was obtained by measuring standards at different concentrations with an injection volume of 10 µl. By the means of this calibration, the amount [mg l^−1^] of guignardic acid, respectively, phenguignardic acid was determined. The regression coefficient amounts to 1.00 for a quadratic curve type.

A Zorbax Eclipse XDB Phenyl-column (250 × 9.4 mm; Agilent, Waldbronn, Germany) with a flow rate of 7 ml min^−1^ was used for preparative HPLC. The compounds were fractionated in an isocratic manner with H_2_O + 0.1% v/v formic acid and acetonitrile.

The mass spectra were recorded by HPLC (Series 1200, Agilent, Waldbronn, Germany) with an UV-DAD and a coupled LC/MSD Trap XCT-ESI-mass spectrometer. An Ascentis^®^ Express C_18_ column (3 × 2.1 mm; 2.7 µm, Supelco, Sigma-Aldrich Chemie GmbH, Steinheim, Germany) was used at 40 °C and a flow rate of 1.0 ml min^−1^. The elution was performed with a gradient of H_2_O + 0.1% v/v formic acid and acetonitrile.

NMR spectra were recorded on an Avance-III 600 MHz spectrometer (Bruker, Rheinstetten, Germany) equipped with a 5 mm cryo-TCI probe head using standard pulse sequences at 23 °C. The signals were referenced to the respective solvent signals of CDCl_3_ (δ_H_ 7.26, δ_C_ 77.16).

### Fermentation

Fermentations were carried out in duplicate. Values given are means of the quantities determined in the individual fermentation experiments. The standard deviation did not exceed 5% ± of the total amount measured. All fermentations were carried out in 1 l malt extract medium composed of malt extract (20 g, Lindenmeyer GmbH & Co. KG, Heilbronn, Germany), peptone (1 g, Becton, Dickinson GmbH, Heidelberg, Germany) and glucose (20 g) per 1 l tap H_2_O in 2 l Erlenmeyer flasks. Agar plugs of well-grown cultures were transferred aseptically as inoculum. Fermentation was carried out at 22 °C on an orbital shaker at 120 rpm. A sample was taken every second day to monitor the production of secondary metabolites and to measure glucose content (Diabur-Test 5000, Roche Diagnostics, Mannheim, Germany) and pH value (pH 209, HANNA^®^ Instruments Deutschland GmbH).

The samples were separated into mycelium and culture filtrate by filtration via a Büchner funnel for HPLC analysis. After extraction of the culture filtrate with an equal volume of EtOAc, the organic solvent was dried over Na_2_SO_4_ and evaporated *in vacuo* to dryness. The mycelium was discarded.

### Microorganisms

Strains examined: CBS 756.70, obtained from the Centraalbureau voor Schimmelcultures (CBS, Fungal Biodiversity Centre, The Netherlands) as *Guignardia aesculi* (Peck) V.B. Stewart, currently valid name *Phyllosticta paviae* Desm. (syn. *Phyllosticta sphaeropsoidea* Ellis & Everh., for phylogeny of this strain see ref. 22). CBS 111645, obtained from CBS as *G. bidwellii* (Ellis) Viala & Ravaz, currently valid name *Phyllosticta ampelicida* (Engelm.) Aa. This strain (cited as ex-type strain^23^) was recently classified as species new to science and called *Phyllosticta parthenocissi* K. Zhang, N. Zhang & L. Cai based on a phylogenetic analysis of combined sequence data of internal transcribed spacer (ITS), translation elongation factor 1-α, glyceraldehyde-3-phospatase dehydrogenase, and actin as well as culture characteristics of this strain. Unfortunately no ecological data are available for this strain^23^. CBS 123405, obtained from CBS as *G. mangiferae* A.J. Roy, currently valid name *Phyllosticta capitalensis* Henn., for a phylogenetic analysis including this strain see ref. 24. CBS 126.22, obtained from CBS as *G. vaccinii* Shear (ex-type), for a phylogenetic analysis including this strain see ref. 23. IMI 147360 (ATCC 28563), obtained from the International Mycological Institute (England) as *G*. *musae* Racib^25^. These fungi were maintained as described by the corresponding culture collection. The principle of ‘one fungus: one name’ was enforced in 2011 (Melbourne Code; ICN) and most *Guignardia* species (sexual forms) relevant for the present study are currently validly cited as species of *Phyllosticta* (former asexual forms; Wulandari *et al*.^18^ and references therein). Nevertheless, the names of the sexual forms are applied in the present document, because they are established as references in this field of study.

### Toxin detection *in planta*

Fresh *G. bidwellii*-infected and non-infected leaf material of *V*. *vinifera* cv. Riesling was analysed for the detection of phytotoxic dioxolanones within infected vine leaf material. Infected (43 g) and non-infected material (50 g), respectively, were transferred to 500 ml EtOAc and shredded by a hand-held blender (ESGE Zauberstab^®^, Unold AG, Hockenheim, Germany). Another 500 ml EtOAc were added and the mixtures were incubated for 30 min at RT on an orbital shaker. After incubation, the extracts were separated from the solids by filtration, the extracts were dried with Na_2_SO_4_ and evaporated *in vacuo* to dryness. Phytotoxic dioxolanones were identified by analytical HPLC and HPLC/MS.

### Toxin production in other species of the genus *Guignardia*

Four other species (phytopathogenic or endophytic) were cultivated, as described above, to examine whether other species of the genus *Guignardia* could produce phytotoxic dioxolanones. The production of secondary metabolites was monitored every second day. The fermentation was stopped when the free glucose was depleted. The culture fluids were extracted, as mentioned above, and secondary metabolites were identified and quantified by analytical HPLC and HPLC/MS.

### Isotopic labelling

*G. bidwellii* was fermented as described above. After the production of known secondary metabolites was detected by analytical HPLC, 1-[^13^C] phenylalanine was supplemented aseptically (final concentration 2.5 mM). Fermentation was stopped as soon as the production rate for the compounds had reached the maximum value. The culture fluid was separated from the mycelium by filtration and was extracted with an equal volume of EtOAc. The dried crude extract was applied onto Chromabond C18ec columns (Macherey-Nagel) and elution was performed in six steps with a gradient of H_2_O + 0.1% v/v formic acid and acetonitrile. The fractions were analysed by HPLC and relevant fractions were purified by preparative HPLC, as described above. Pure compounds of guignardic acid, phenguignardic acid and alaguignardic acid were analysed by NMR spectroscopy for ^13^C enrichment.

## Electronic supplementary material

Supporting information
