# Supplementary material for: Phytotoxic dioxolanones are potential virulence factors in the infection process of Guignardia bidwellii
Source: Sci Rep. 2017 Aug 21;7:8926. doi: 10.1038/s41598-017-09157-6 (PMC5566483; doi:10.1038/s41598-017-09157-6)
Supplement: Supplementary file 1 — Supporting information [file 41598_2017_9157_MOESM1_ESM.pdf]

Supporting information

Phytotoxic dioxolanones are potential virulence factors in the infection process of *Guignardia bidwellii*

Buckel, Iris; Andernach, Lars; Schüffler, Anja; Piepenbring, Meike; Opatz, Till; Thines, Eckhard

Toxin detection *in planta*

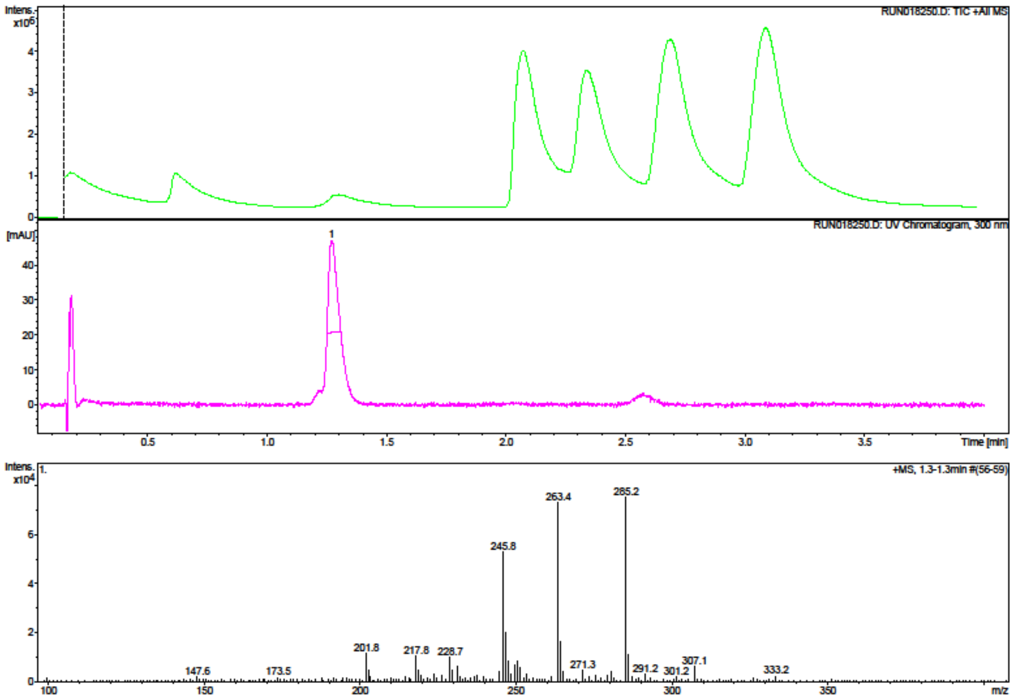

Fig. 1: HPLC-ESIMS run data of the HPLC peak (retention time 8.7 min). (gradient: 70:30 -> 65:35 H_2O/MeCN in 4 minutes, then in 2 minutes 65:35 -> 10:90 H_2O/MeCN)

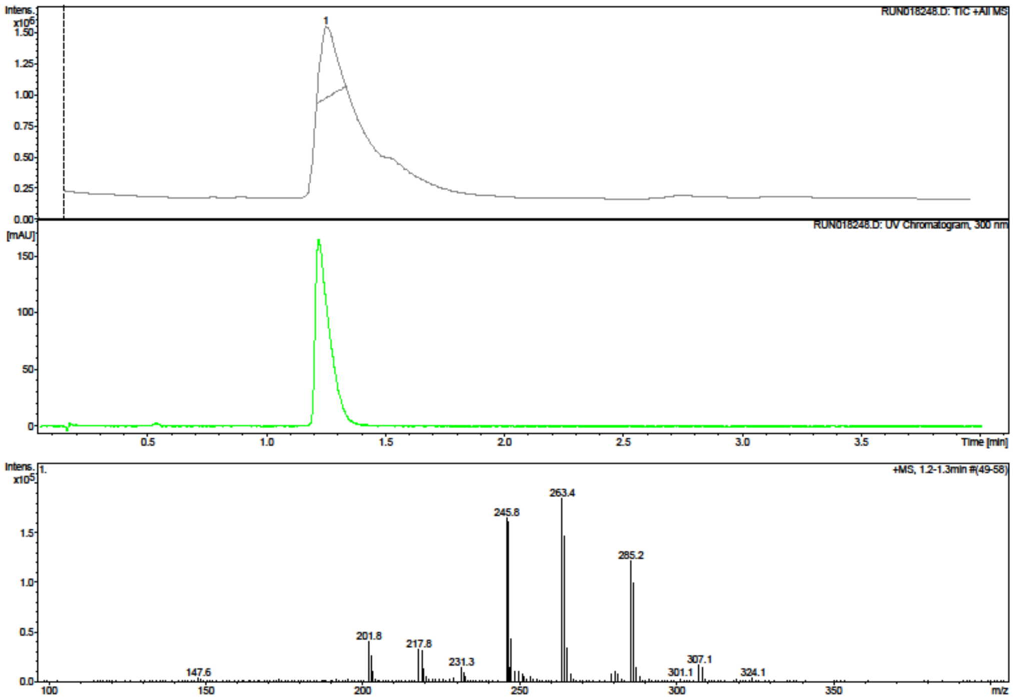

Fig 2: HPLC-ESIMS data of isolated partially ^{13}C-labeled guignardic acid. (gradient: 70:30 -> 65:35 H_2O/MeCN in 4 minutes, then in 2 minutes 65:35 -> 10:90 H_2O/MeCN)

Print of window 39: UV Apex spectrum of Peak 1.22 of RUN018250.D

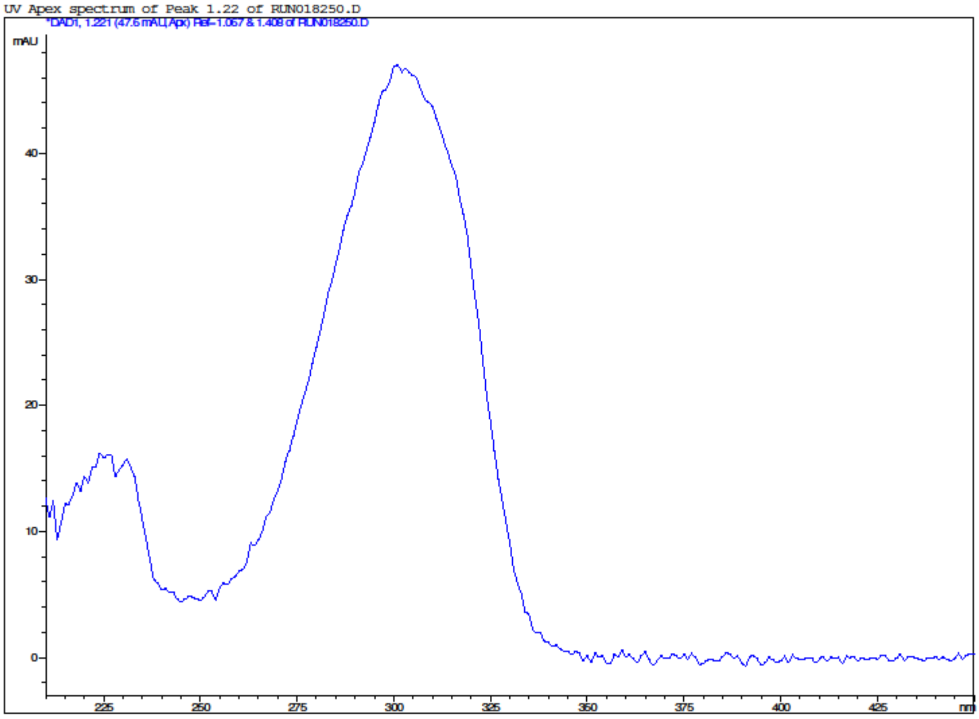

Instrument 1 2/23/2016 5:23:27 PM NN   Page 1 of 1

Fig. 3: UV spectrum at 1.3 minutes of the collected HPLC peak (retention time 8.7 minutes).

Print of window 39: UV Apex spectrum of Peak 1.168 of RUN018248.D

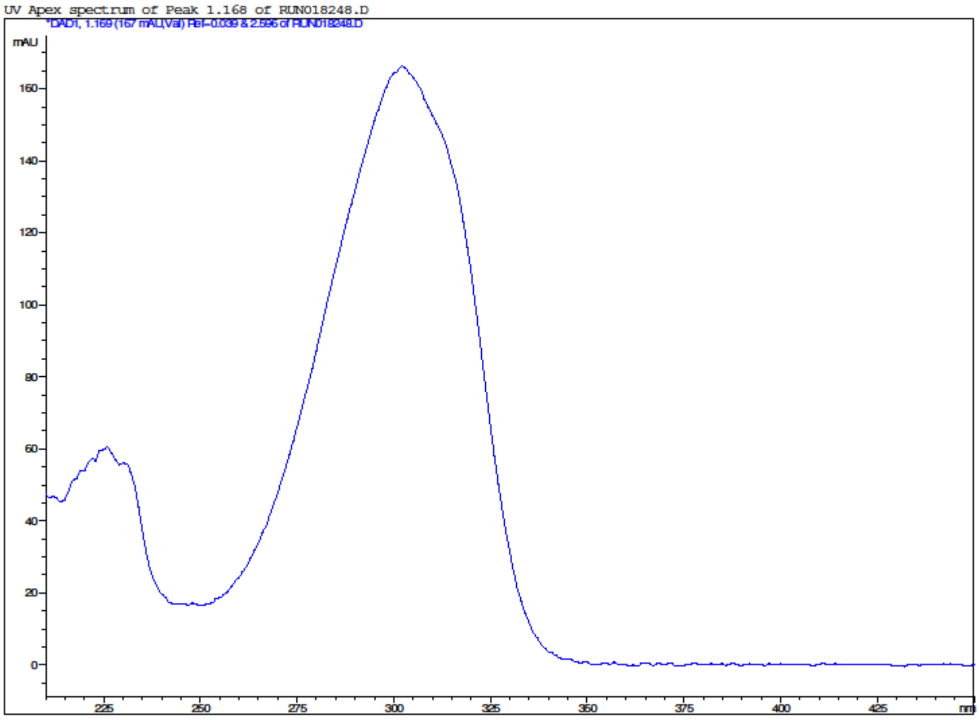

Instrument 1 2/23/2016 5:20:33 PM NN   Page 1 of 1

Fig. 4: UV spectrum at 1.3 minutes of isolated partially ^{13}C-labeled guignardic acid.

<u>Elucidation of the precursor for the biosynthesis of phytotoxic dioxolanones</u>

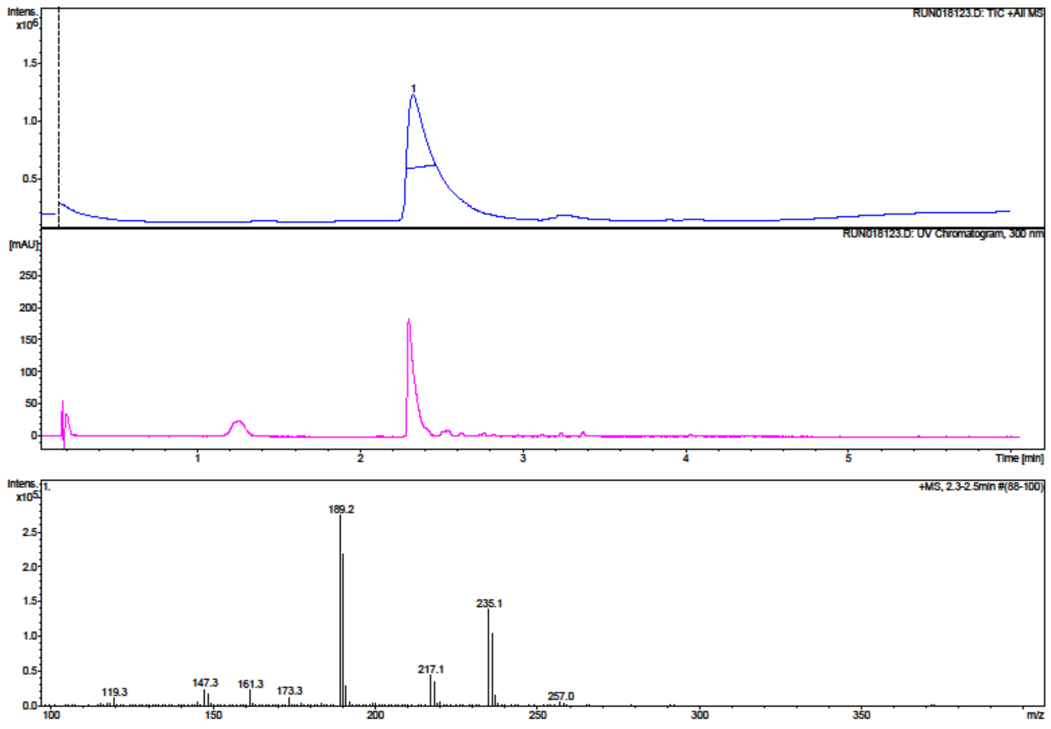

Fig. 5: HPLC-ESIMS data of isolated partially ^{13}C-labeled alaguignardic acid. (gradient: 90:10 -> 10:90 H_2O/MeCN in 4 minutes, then 2 minutes 10:90 H_2O/MeCN)

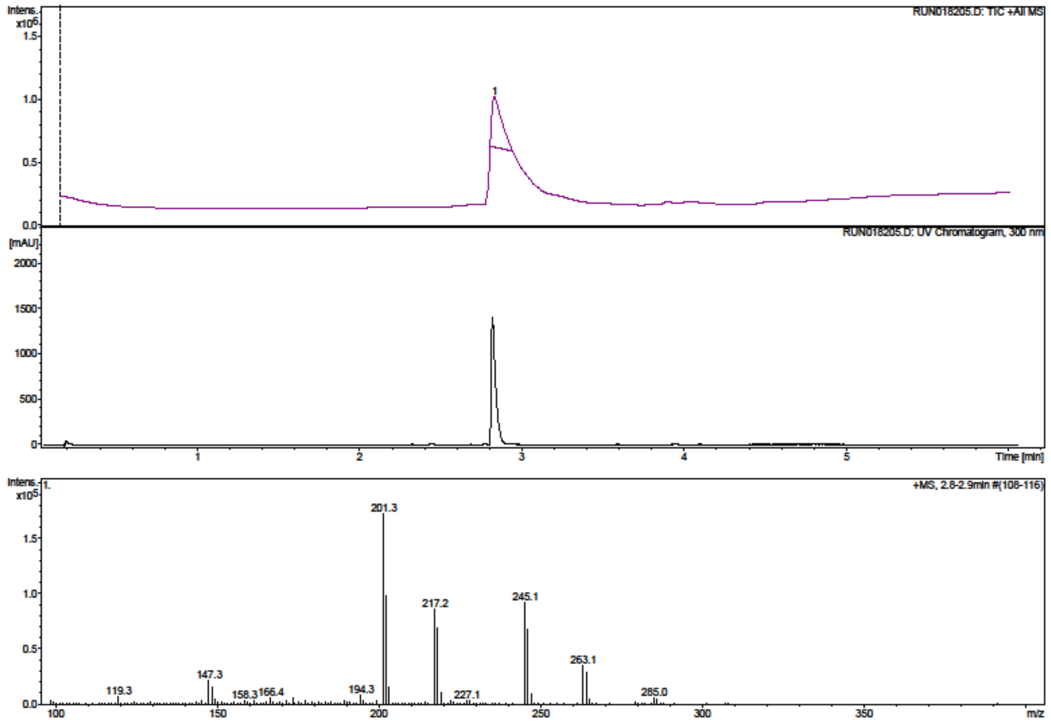

Fig. 6: HPLC-ESIMS data of isolated partially ^{13}C-labeled guignardic acid. (gradient: 90:10 -> 10:90 H_2O/MeCN in 4 minutes, then 2 minutes 10:90 H_2O/MeCN)

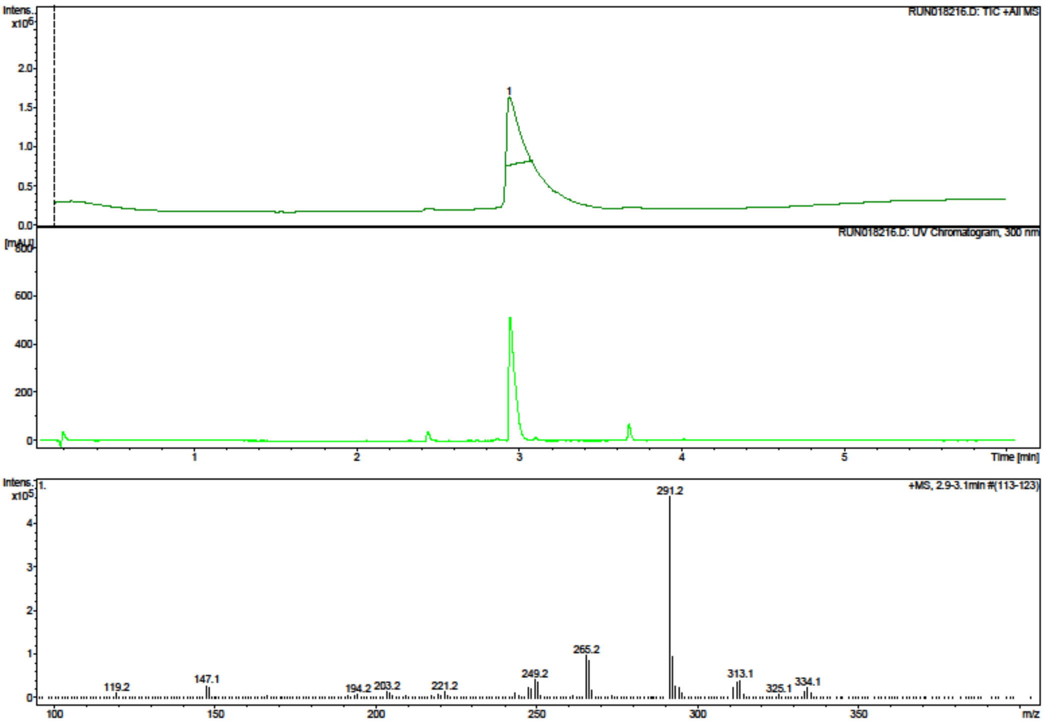

Fig. 7: HPLC-ESIMS data of isolated partially ^{13}C-labeled phenguignardic acid. (gradient: 90:10 -> 10:90 H_2O/MeCN in 4 minutes, then 2 minutes 10:90 H_2O/MeCN)

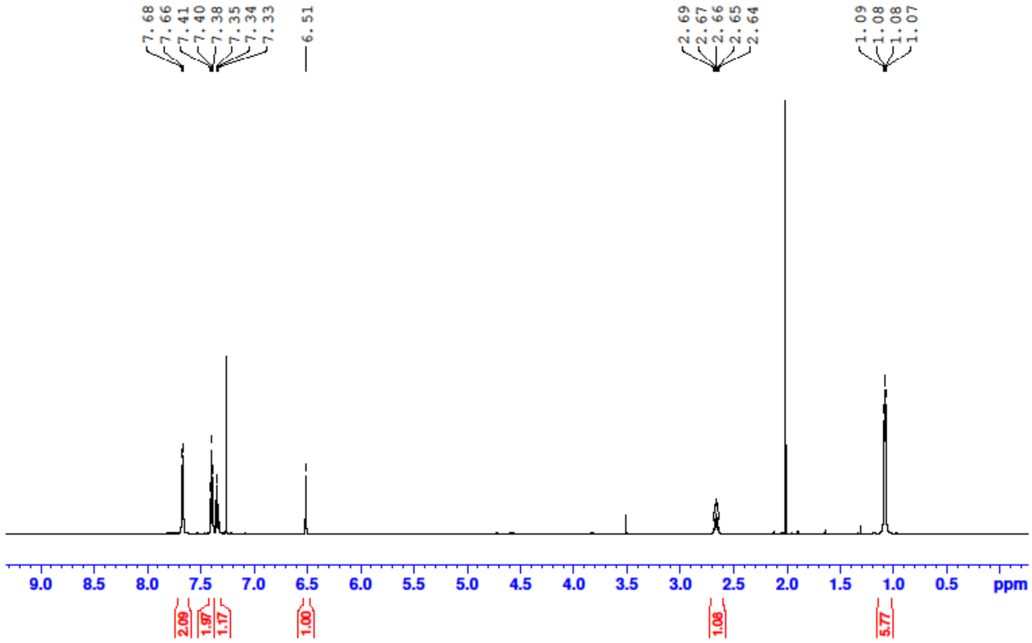

Fig. 8: ^{1}H-NMR of partially ^{13}C labeled guignardic acid (600 MHz, $CDCl_3$).

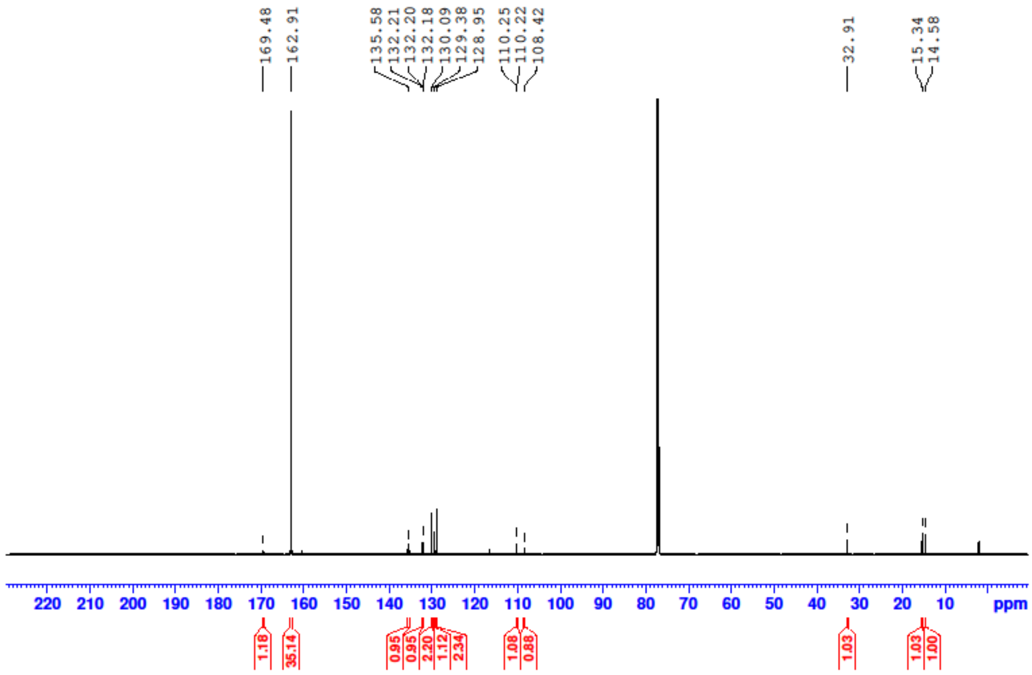

Fig. 9: Igated ^{13}C-NMR of partially ^{13}C labeled guignardic acid (151 MHz, $CDCl_3$).

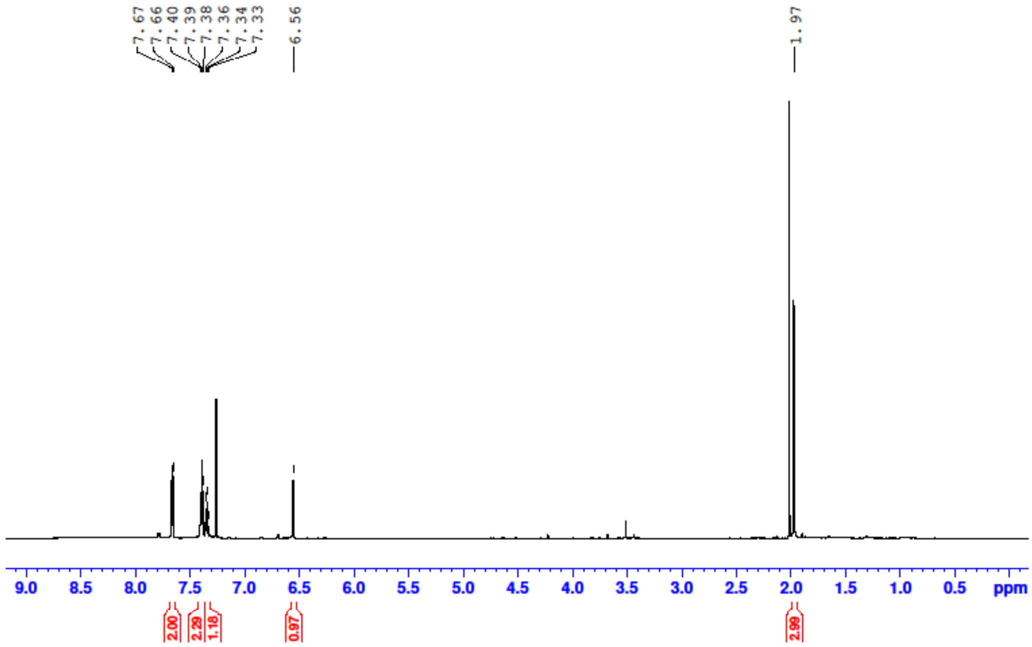

Fig. 10: ^{1}H-NMR of partially ^{13}C labeled alaguignardic acid (600 MHz, $CDCl_3$).

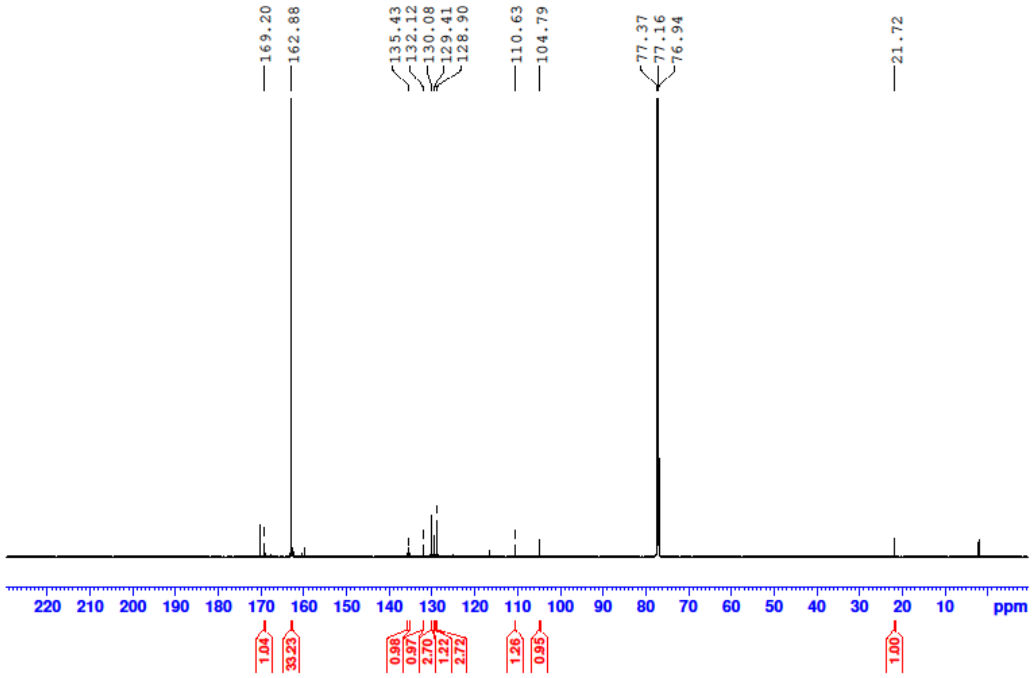

Fig. 11: Igated ^{13}C-NMR of partially ^{13}C labeled alaguignardic acid (151 MHz, $CDCl_3$).

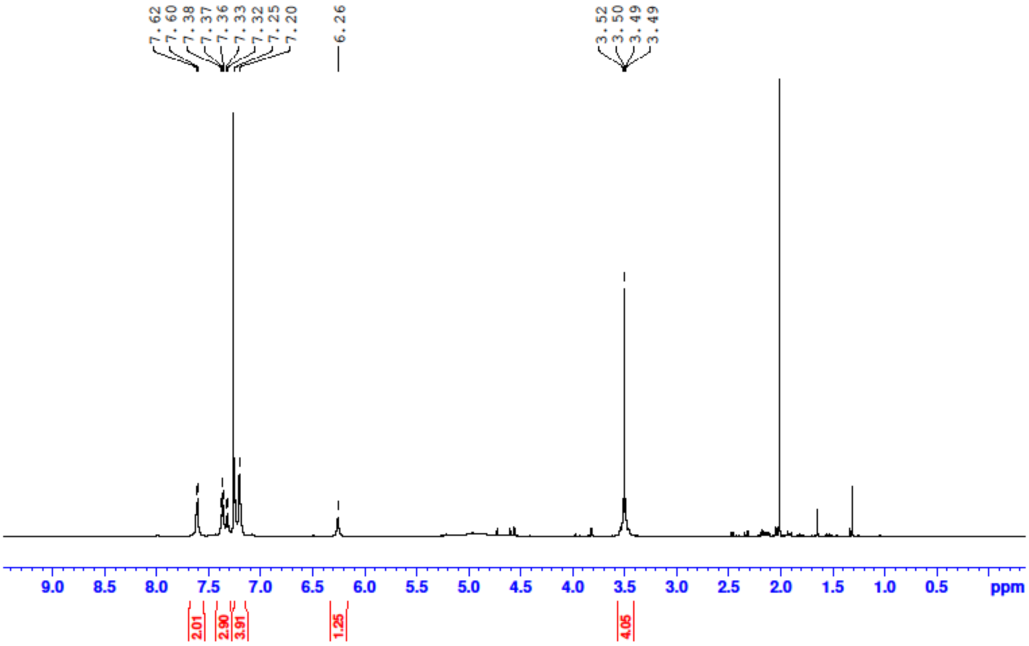

Fig. 12: ^{1}H-NMR of partially ^{13}C labeled phenguignardic acid (600 MHz, $CDCl_3$).

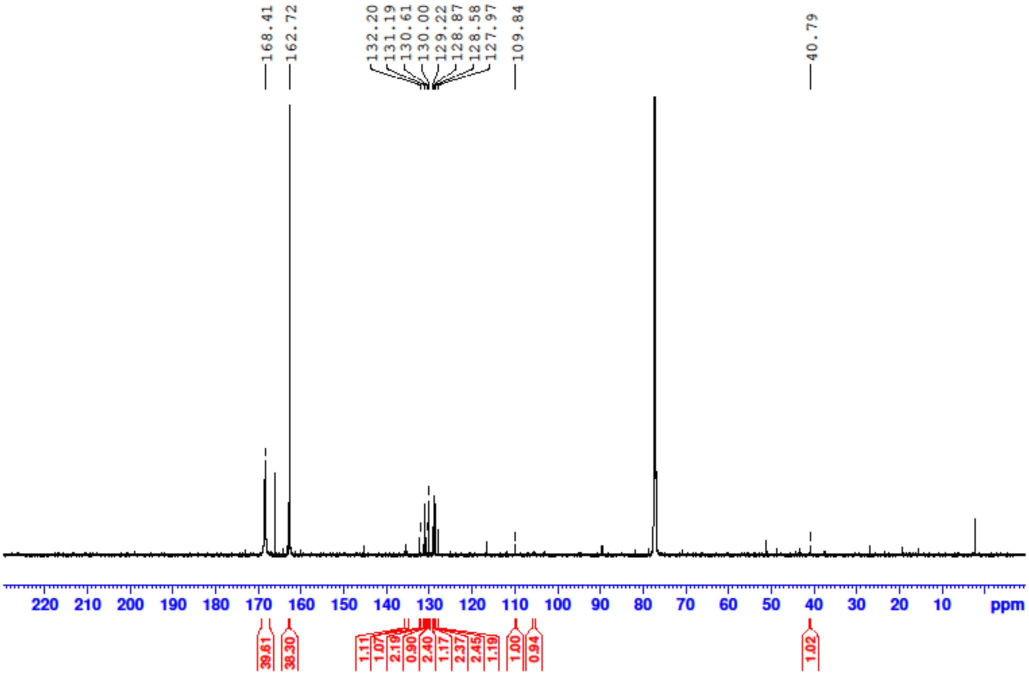

Fig. 13: Igated ^{13}C-NMR of partially ^{13}C labeled phenguignardic acid (151 MHz, $CDCl_3$).
